# Evaluation of Shifted Excitation Raman Difference Spectroscopy and Comparison to Computational Background Correction Methods Applied to Biochemical Raman Spectra

**DOI:** 10.3390/s17081724

**Published:** 2017-07-27

**Authors:** Eliana Cordero, Florian Korinth, Clara Stiebing, Christoph Krafft, Iwan W. Schie, Jürgen Popp

**Affiliations:** 1 Leibniz Institute of Photonic Technology (IPHT), Albert-Einstein-Straße 9, Jena 07743, Germany; eliana.cordero@leibniz-ipht.de (E.C.); florian.korinth@leibniz-ipht.de (F.K.); clara.stiebing@leibniz-ipht.de (C.S.); christoph.krafft@ipht-jena.de (C.K.); juergen.popp@leibniz-ipht.de (J.P.); 2 Institute of Physical Chemistry and Abbe Center of Photonics, Friedrich Schiller University Jena, Helmholtzweg 4, Jena 07743, Germany

**Keywords:** Raman spectroscopy, SERDS, EMSC, background correction, signal to noise ratio

## Abstract

Raman spectroscopy provides label-free biochemical information from tissue samples without complicated sample preparation. The clinical capability of Raman spectroscopy has been demonstrated in a wide range of in vitro and in vivo applications. However, a challenge for in vivo applications is the simultaneous excitation of auto-fluorescence in the majority of tissues of interest, such as liver, bladder, brain, and others. Raman bands are then superimposed on a fluorescence background, which can be several orders of magnitude larger than the Raman signal. To eliminate the disturbing fluorescence background, several approaches are available. Among instrumentational methods shifted excitation Raman difference spectroscopy (SERDS) has been widely applied and studied. Similarly, computational techniques, for instance extended multiplicative scatter correction (EMSC), have also been employed to remove undesired background contributions. Here, we present a theoretical and experimental evaluation and comparison of fluorescence background removal approaches for Raman spectra based on SERDS and EMSC.

## 1. Introduction

There is a significant requirement for rapid and minimally to non-invasive tools for cancer diagnosis. The ability to obtain specific biochemical information from biological samples without the need for labeling makes Raman spectroscopy attractive for many diagnostic applications in medicine [1,2]. For instance, Raman spectroscopy has been used to accurately identify and grade transitional cell carcinoma (TCC) in vitro and has shown promising results as an auxiliary method for pathological identification of bladder tissue [3,4,5,6]. The development of optical fiber Raman probes with high efficiency and throughput provided the opportunity to perform in vivo measurements of skin, brain, esophagus, and bladder, demonstrating that data acquisition, analysis and diagnostics can be performed at the patient in real time [7,8,9,10].

Human tissue is mainly composed of proteins, lipids, nucleic acids, and carbohydrates [11]. One of the main challenges for the implementation of Raman spectroscopy in the clinic is the excitation of auto-fluorescence in tissue. Biological tissues can show significant auto-fluorescence [12], which depends on the excited fluorophores, the excitation wavelength, and is several magnitudes stronger than the Raman signal, leading to high shot noise. A considerable number of instrumental [13,14,15,16] and computational methods have been proposed to reduce or subtract the fluorescence background contribution in Raman spectra [17,18,19,20].

Computational methods include, for example, polynomial fitting [21], different least squares methods [22] and extended multiplicative scatter correction (EMSC) [23]. Polynomial fitting approximates the broad fluorescence background as an n-order polynomial function, fitting the polynomial function to spectral areas without Raman bands. Next to manually deciding which areas should be used, automatic methods for curve fitting in chromatographic analysis have been proposed under high noise circumstances, which is a limiting factor for this method [24]. Least squares methods rely on fitting of linear combinations of reference data to the measured spectra, where the sum of the squared differences between the observed values and the fitted values is minimized [25]. Different least squares approaches have previously been implemented, such as for instance iterative least squares or weighted least squares [26,27]. One very promising framework for model-based background correction in Raman spectroscopy is EMSC [28]. It is also based on a least squares fitting of pre-defined background spectra, but also including the fitting of pure components spectra [23]. Further computational techniques used to separate the fluorescence background from Raman spectra are principal component analysis [29], and wavelets analysis [30]. Wavelets analysis decomposes the spectrum into different frequency components and the background is suppressed by setting the very low frequency components to zero. Furthermore, by setting the high frequency components to zero as well, noise contributions can be removed. However, the transformation of the signal into frequency bands may result in distortion in some spectral areas [19,31].

As a methodical approach photobleaching has been suggested to remove fluorescence. By irradiating the sample with an excitation laser for a long period of time, a photolytic decomposition of the fluorescent interfering molecules is induced [14,15,32]. Another technique that addresses the reduction of the fluorescence background, as well as low signal to noise ratio (SNR) problems, is time-gating Raman spectroscopy. It employs ultra-short laser pulses that drive an all-optical Kerr shutter to gate early-arriving Raman photons, while blocking the later-arriving fluorescence photons. However, the systems are highly complex, costly, and challenging to modify [33]. Modulated excitation wavelength shifting is an instrumental method, where Raman measurements are performed at two closely spaced excitation wavelengths to obtain two shifted Raman spectra. By introducing a low-frequency modulator, a Raman spectrum is reconstructed. The main limitation is the need of lock-in detection [34]. Modulated Raman spectroscopy was introduced with a frequency-modulated excitation laser, continuous acquisition of Raman spectra, and without lock-in detection [35]. A popular instrumental method that has been widely applied is shifted excitation Raman difference spectroscopy (SERDS) [16,36,37,38,39]. SERDS is based on the principle that small changes in the excitation wavelength result in a spectral shift of the Raman spectrum, while the fluorescence contribution does not change spectrally. By calculating the difference between closely shifted Raman spectra all wavelength-independent contributions are removed. It was shown that not only fluorescence can be effectively removed by this method, but also ambient light, etaloning, and any other non-varying source [40,41,42]. Since the positions of the Raman bands are used for the interpretation of Raman spectra, it was previously suggested that the optimal shift in excitation wavelength should correspond to the full width at half maximum of a relevant Raman band [43,44]. Applying this shift results in a difference spectrum were the band position corresponds to the inflection point between the minimum and maximum of the respective band. This assumption is valid for samples, which have spectral bands with similar spectral widths, such as powders or crystalline proteins, and are acquired at very high spectral resolution. Since the Raman bandwidth of specific biological components vary within one spectrum, this theoretical optimal shift for the reconstruction of band positions cannot be applied for complex biological Raman spectra. Moreover, for biomedical applications there is not just one single Raman band, which is important for the differentiation between, e.g., tumor and non-tumor tissues.

Previous studies have evaluated the capability of SERDS to subtract strong fluorescence background exceeding the Raman signal up to a ratio of 200:1 [36,44,45,46,47,48]. A main topic in these studies is the necessary processing of SERDS spectra, using computational methods in order to acquire typical Raman spectra [37,49,50,51]. However, there are no studies where SERDS is evaluated and compared directly to computational background correction methods.

In this study, theoretical and experimental results for SERDS spectra of biological components, such as lipids and proteins, are evaluated. SNRs are determined based on different fluorescent background estimations. Additionally the concept of an optimal shift for SERDS is discussed theoretically and experimentally. Different algorithms for optimizing the spectral quality of the difference spectra are tested and compared to EMSC as a conventional computational background correction method.

## 2. Materials and Methods

### 2.1. Theoretical Approach

**Figure 1:**
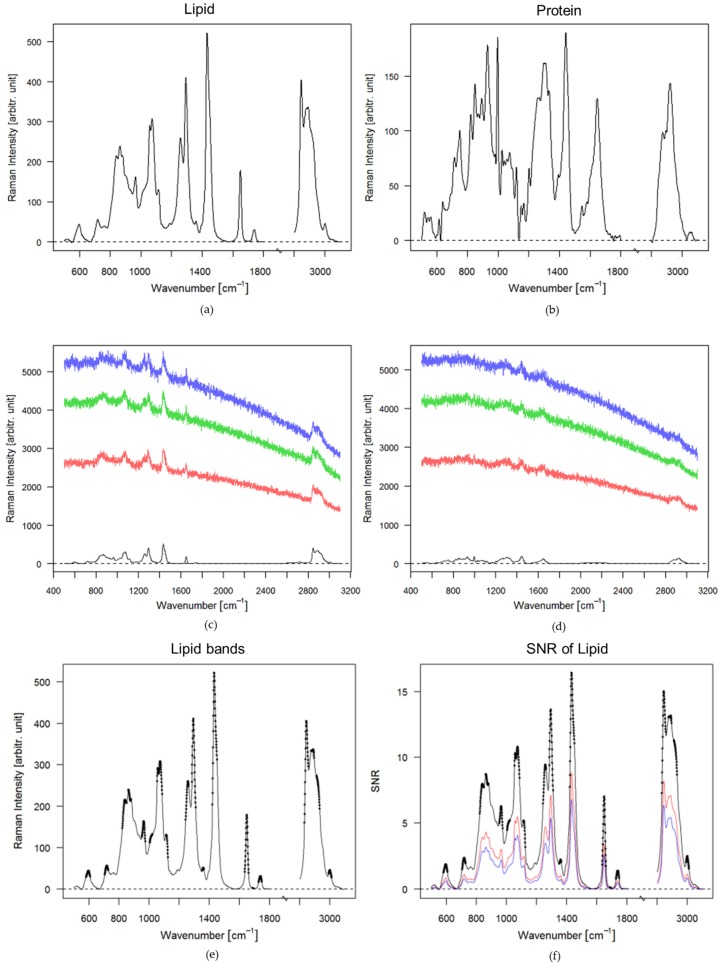
Baseline corrected spectra and spectra with simulated fluorescence backgrounds and shot noise levels: (**a**) Baseline corrected spectrum of lipids; (**b**) Baseline corrected spectrum of proteins; (**c**) Lipid spectra with added fluorescence intensities, i.e., 10 (blue), 8 (green) and 5 (red) times the maximal band of the lipid signal, including corresponding noise levels. The black spectrum is the lipid spectrum without fluorescence; (**d**) Protein spectra with added fluorescence intensities, i.e., 10 (blue), 8 (green) and 5 (red) times the maximal band of the lipid signal, including corresponding noise levels. The black spectrum is the protein spectrum without fluorescence; (**e**) Spectral bands and windows of the measured lipid spectrum, the black points are the maxima and the windows estimated by the Savitzky and Golay function; (**f**) SNR of the lipid spectra with fluorescence, the black spectrum is the SNR of the lipid spectrum, the red spectrum correspond to the SNR of the lipid spectrum with fluorescence 5 times the maximal band of the lipid signal, the blue spectrum is the SNR of the lipid spectrum with fluorescence factor 10 of the lipid signal.

Raman spectra of lipid and protein components of chicken meat with a low fluorescence background were recorded, using a commercial Raman microscope setup (Holoprobe, Kaiser Optical System, Ann Arbor, MI, USA) with an excitation wavelength of 785 nm and an integration time of 1 s, see Figure 1a,b. These spectra were used as a basis for the theoretical analysis on the influence of fluorescent background contributions and noise levels on the recovery of informational content.

Data analysis of the Raman spectra and spectral simulations were performed in R [52], using the packages hyperSpec, Ramancal, baseline, and pracma [53,54,55,56]. The recorded Raman spectra were wavelength calibrated, during which all spectra were corrected to the same wavenumber axis relative to 785 nm. Furthermore, the spectra were background corrected by using support points determined from a convex hull of the spectrum and smoothed by applying a local polynomial regression fitting. The processed spectra were shifted by multiple wavelength steps between 1 nm and 4 nm to simulate Raman spectra recorded at different excitation wavelengths. The optimal shift was evaluated using the autocorrelation function from the forecast package [57,58]. The computational background correction was performed on the measured Raman spectra at 785 nm excitation, using the emsc function available in the cbmodels package [59]. For the simulations of different auto-fluorescence contributions, fluorescence spectra were measured on the commercial Raman setup and fitted by a polynomial function. The different fluorescence intensity levels were simulated by the multiplication of a constant factor and added to the Raman spectra of lipids and proteins. The noise contribution was estimated by applying a Poisson distribution function on the square root of the intensity, see Figure 1c,d.

SNRs were determined under the shot noise-limited definition [60], where the noise is calculated as the square root of the signal intensity. The SNR was calculated for each pixel of the spectrum, leading to a wavenumber-dependent SNR spectrum. Regions without relevant bands were removed. To determine a single SNR value for each spectrum, the second derivative of the spectrum without noise was calculated and the maxima including surrounding points of a certain wavenumber window were detected. A threshold was used to determine spectral locations to calculate the SNRs of importance, see Figure 1e,f. Those selected SNRs were averaged to obtain a mean SNR for the entire spectrum. The second derivative and smoothing of the preprocessed spectrum were performed by using the polynomial filtering method of Savitzky and Golay [55].

### 2.2. Experimental Approach

Raman measurements were performed using a tunable diode laser DL Pro (Toptica Photonics AG, Gräfelfing, Germany) enabling a tuning range from 765 to 805 nm. To achieve a higher output power the laser was coupled, using a single mode fiber, into a laser amplifier (BoosTA, Toptica Photonics AG). The output laser power at 785 nm after the amplifier was adjusted to 200 mW. The laser power at 785 nm after the objective was 95 mW. No damage to the tissue was observed.

Excitation wavelengths between 784 and 786 nm where chosen. Wavelengths below and higher this range experienced high losses due to filter parameters, resulting in reduced excitation power and lower Raman signals. The laser was guided into a home-built microscope setup with a 40× magnification, NA = 0.55 objective lens. The setup has been previously described [61]. The sample was placed on a holder, which is mounted on two motorized x-y translational stages (CONEX MFA-Series; Newport, Irvine, CA, USA). To allow a sample translation in z-direction the motorized x–y translational stages were mounted on an automated z-positioning stage (MTS25-Z8, Thorlabs, Newton, NJ, USA). The generated Raman signal was collected by the same objective lens and separated from the excitation light by a dichroic notch filter (785 nm, bandwidth 89 nm; Semrock, Rochester, NY, USA). An additional notch filter (785 nm ± 19 nm; Laser Components, Olching, Germany) was used for a reliable suppression of the excitation light. An achromatic doublet (100 mm; Thorlabs) focuses the Raman signal onto a multimode fiber (105 μm core, Thorlabs). The signal was then fiber-coupled into a spectrometer (IsoPlane160, Princeton Instruments, Acton, MA, USA) that is equipped with a grating with 400 grooves/mm, blazed at 750 nm, and allows a spectral resolution of 9 cm^−1^. The signal is dispersed onto a charge-coupled device (CCD) (PIXIS-400BR-eXcelon; Princeton Instruments) with a nominal quantum efficiency of up to 98% at 800 nm. The setup is controlled by in-house written data-acquisition software in LabView (National Instruments, Austin, TX, USA). The data acquisition was designed such that at each spatial location in the sample five Raman spectra at different excitation wavelengths, ranging between 784 nm to 786 nm with an interval of 0.5 nm, were measured consecutively. After all measurements for one spatial location were performed, the next location was measured.

Raman images were acquired of meat samples, providing typical biological spectra of fibrous protein from muscle tissue and tendons, non-fibrous proteins, lipids, and bone. Images were acquired of 10 mm by 10 mm areas with a step size of 0.5 mm, and an integration time of 1 s. Data analyses were performed in R [52]. All spectra were cleared from cosmic spikes [62,63]. To generate background corrected Raman spectra, the spectra measured at 785.0 nm were baseline corrected by EMSC. Seven components, i.e., constant offset, a linear function, and multiple fluorescence functions were used as background estimates; and Raman spectra of collagen, protein, lipid spectrum and bone were used for pure component spectra. The components were fitted to the measured Raman spectra. The determined coefficients multiplied with the background component spectra were subtracted from the raw spectra. The corrected spectra were area normalized and grouped into four clusters by hierarchical cluster analysis (HCA).

Difference spectra were obtained by subtracting the Raman spectra acquired at different excitation wavelengths from the Raman spectrum measured at the excitation wavelength of 786 nm of the same spatial location in the tissue sample. Thereby, four SERDS spectra per image point with different wavelength shifts, i.e., 0.5 nm, 1.0 nm, 1.5 nm, 2.0 nm, were obtained, which can be represented in four SERDS images containing 400 Raman difference spectra. Before calculating the differences spectra, the cosmic spike-corrected spectra were normalized or optimized. Three different types of processing methods were tested:

(1) $$\mathrm{area}\;\mathrm{normalization}:S_{an}\left({\tilde{\nu }}_{i}\right)=\frac{S\left({\tilde{\nu }}_{i}\right)}{\sum_{i=1}^{n}S\left({\tilde{\nu }}_{i}\right)}$$

z-score normalization/standard normal variate [64]:

(2) $$S_{zn}\left({\tilde{\nu }}_{i}\right)=\frac{S\left({\tilde{\nu }}_{i}\right)-mean\left(S\right)}{SD\left(S\right)}$$

(3) $$\mathrm{subtraction}\;\mathrm{optimization}:minimization\;of\;AUC\;\left(\left|S_{1}-\left(x\cdot S_{2}\right)\right|\right)$$

with *S*—signal; $\tilde{\nu }$—relative wavenumber; *SD*—standard deviation; *AUC*—area under the curve.

For the subtraction optimization a random value *x* is multiplied to the spectrum, which is being subtracted, and the area under the curve (*AUC*) of the absolute values of the difference spectrum is calculated. The value *x* is than iteratively adjusted, so that the *AUC* is minimized.

## 3. Results

### 3.1. Theoretical Approach

Ideally, Raman spectra show defined bands, as seen in Figure 1a for lipids and Figure 1b for proteins. However, due to the high auto-fluorescence background in biological tissue, Raman bands are superimposed with an auto-fluorescence background and as well as accompanying shot noise, shown in Figure 1c,d.

Even small amounts of fluorescent molecules in a sample can cause high background intensities. The fluorescence scattering cross-section is usually several orders of magnitude higher than the Raman scattering cross-section [65,66].

After adding the simulated fluorescence background and shot noise to the Raman signal, as shown in Figure 1, the Raman bands can barely be distinguished, because the noise level is increasing with the intensity of the fluorescence signal, following the square root of the total signal intensity. Therefore, at higher fluorescence contributions the SNR is lower for a constant Raman signal intensity, as shown in Figure 1f. An estimated average SNR value of the main bands for each spectrum was calculated based on the described algorithm in the materials and method Section 2.1. The average SNR for the lipid spectrum without fluorescence is approximately 6.8 (Figure 1f, black spectrum). If the lipid spectrum has a fluorescence intensity five times higher than the maximal band intensity of the lipid spectrum the average SNR decreases to 3.4 (Figure 1f, red spectrum). For a fluorescence intensity 10 times higher than the maximal band intensity of the lipid spectrum the average SNR is only 2.5.

**Figure 2:**
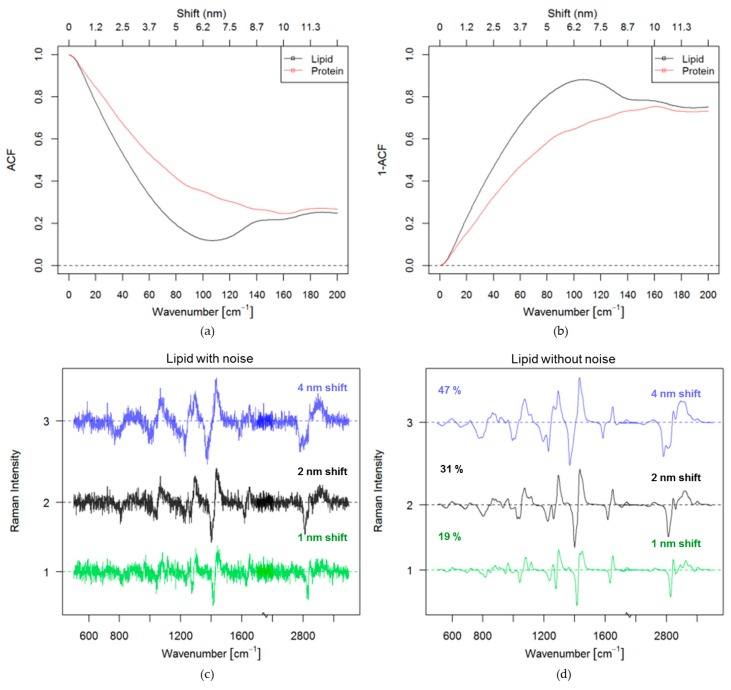
Optimal shift: (**a**) Estimation by using the autocorrelation function of lipid and protein spectra (**b**) Estimation by using the 1-autocorrelation function of lipid and protein spectra; (**c**) Simulation of the difference spectra of lipid at 1 nm shift (green), 2 nm shift (black) and 4 nm shift (blue) with noise, the values are the percentage values of the retained signal; (**d**) Simulation of the difference spectra of lipid at 1 nm shift (green), 2 nm shift (black) and 4 nm shift (blue) without noise.

In order to evaluate different methods to correct auto-fluorescence background contributions a set of fluorescence intensities was created to simulate fluorescence contributions at different intensities and hence, different SNRs for a Raman signal with constant signal intensity. SERDS simulations were then performed on the set of created noise and background affected spectra. Before the evaluation of SERDS for background correction, an optimal laser wavelength shift had to be determined. The optimal shift distance has often been defined as the wavelength difference, which corresponds to the half-width of a Raman band [43]. However, this is only valid when bands with similar bandwidth are present within a spectrum, which is not the case in Raman spectra of most biological samples as discussed in the introduction. Therefore, the optimal wavelength shift was defined as the shift, which retains most of the signal information in the difference spectrum. Autocorrelation of pure Raman spectra were performed to determine the optimal shift. The autocorrelation function of lipid (black) and protein (red) is shown in Figure 2a.

A complete overlap between the spectrum and itself results in a perfect autocorrelation (acf = 1), seen in Figure 2a. Meaning, if two signals are fully overlapped the difference spectrum of those signals will have zero informational content. A very low autocorrelation coefficient indicates a low self-similarity of the signal and will result in high signal intensities in the difference spectra. Hence, high 1-acf values will result in higher signal of difference spectra. This approach was used as an indicator for the optimal shift for pure spectra of lipid and protein. Shifts were analyzed in a region between 0 to 12.6 nm because a higher shift might result in effects on the fluorescence spectrum and are technically very challenging to implement. The highest intensities retained in the difference spectra were for a shift of 7 nm for lipid, and 10 nm for protein, corresponding to 110 and 160 cm^−1^, respectively (see Figure 2b). These values are much higher than commonly reported [37,67,68]. It is also visible in Figure 2b that when the shift in wavelength for lipid is higher than 7 nm the 1-acf decreases, resulting in a reduction of signal. The influence of the shift on the retained signal intensity can be best visualized by plotting the corresponding difference Raman spectra for lipids with and without noise, as seen in Figure 2c,d. For a shift of 1 nm (green) there is 47% of the signal retained, for a shift of 2 nm (black) 54% of the signal is retained and by shifting 4 nm (blue) a considerable part of the signal is retained (68%).

**Figure 3:**
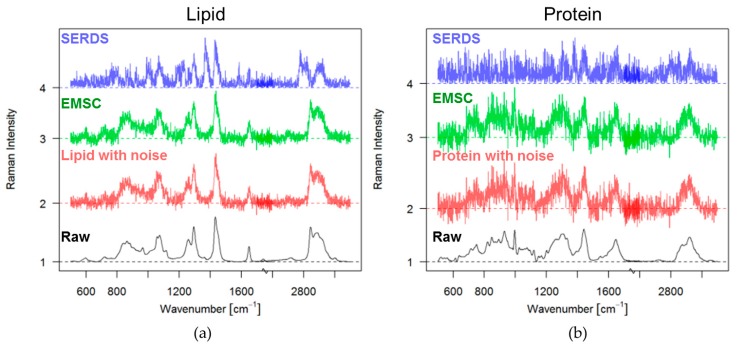
EMSC and SERDS spectra for lipid and protein: (**a**) Lipid spectrum without background (black); lipid spectrum with noise added (red, noise corresponds to the poison distribution of original spectrum and the added fluorescence five times the maximal band of the lipid signal); EMSC corrected lipid spectrum after adding a fluorescence background five times the maximal band of the lipid signal (green); absolute values of SERDS difference spectrum of lipid after shifting 4 nm (blue); (**b**) Protein spectrum without background (black); protein spectrum with noise added (red, this noise corresponds to the poison distribution of original spectrum and the added fluorescence five times the maximal band of the protein signal); EMSC corrected protein spectrum after having fluorescence background five times the maximal band of the protein signal (green); absolute values of SERDS difference spectrum of protein after shifting 4 nm (blue).

To compare SERDS and EMSC the simulated Raman spectra with fluorescence and noise from Figure 1c,d, with a fluorescence intensity level of five times the maximal band of the lipid signal, were chosen. A Raman shift of 4 nm was used and after applying SERDS the absolute values of the difference lipid and protein spectra were calculated (see Figure 3a,b, blue spectra).

The absolute values of the difference spectra are illustrated in order to indicate how much signal is retained in the difference spectra. The same plot also shows the EMSC corrected spectra (green), and the original Raman spectra with and without noise, colored red and black, respectively. The processed SERDS signal (blue) has lower intensity than the EMSC corrected spectra (green). When comparing the EMSC corrected spectra (green) with the original spectra with noise (red) in Figure 3, one can see that it resembles the original spectra with noise quite well. The intensity of the signal is not reduced as in the case of the difference spectrum based on SERDS (blue). The reader should keep in mind that the acquisition time for SERDS is twice as long, as for a normal spectrum, because two spectra are acquired. Hence, the intensity of the Raman signal for the background corrected case should actually be twice as high.

In a shot noise limited measurement the SNR of a Raman signal is inversely proportional to the square root of the sum of the Raman signal and the fluorescence (Equation (4)). Consequently, the SNR will be very low at very high fluorescence intensities. For SERDS a further problem occurs, which is that while the signal intensity is subtractive, the noise contribution is additive, Equation (5). Hence, one disadvantage in SERDS is the reduction of the overall SNR in the difference spectra. It is possible to express the SNR of SERDS measurements in terms of the SNR of Raman measurements for identical acquisition conditions, i.e., the acquisition time of a Raman spectrum has to match the acquisition time of two SERDS spectra.

Equation (9) shows that for *S*_2_ smaller than *S*_1_ the *SNR_SERDS_* will be smaller or equal to half of the *SNR_RAMAN_*; for *S*_2_ = *S*_1_ *SNR_SERDS_* is 0; and for *S*_2_ ≥ 2*S*_1_ *SNR_SERDS_* ≥ *SNR_RAMAN_*/2. The last term indicates that the SERDS difference spectrum can locally have a higher SNR as a Raman spectrum. However, in general the total spectral SNR is of importance and the local SNR is not always meaningful. The summation in Equation (10) allows assessing the SNR over the entire measurable spectral region or a region of interest. Because the autocorrelation function for any reasonable shift is non-zero (see also Figure 2a), the total SNR of SERDS is always smaller than the SNR of Raman.

(4) $$SNR_{RAMAN}\left(\lambda_{i}\right)=\frac{S_{1}\left(\lambda_{i}\right)}{\sqrt{S_{1}\left(\lambda_{i}\right)+F_{1}\left(\lambda_{i}\right)}}$$

(5) $$SNR_{SERDS}\left(\lambda_{i}\right)=\left|\frac{S_{1}\left(\lambda_{i}\right)-S_{2}\left(\lambda_{i}\right)}{\sqrt{S_{1}\left(\lambda_{i}\right)+F_{1}\left(\lambda_{i}\right)+S_{2}\left(\lambda_{i}\right)+F_{2}\left(\lambda_{i}\right)}}\right|$$

for S≪F and $F_{1}\left(\lambda_{i}\right)\approx F_{2}\left(\lambda_{i}\right)$

(6) $$SNR_{SERDS}\left(\lambda_{i}\right)=\frac{1}{\sqrt{2}}\left|\frac{S_{1}\left(\lambda_{i}\right)-S_{2}\left(\lambda_{i}\right)}{\sqrt{S_{1}\left(\lambda_{i}\right)+F_{1}\left(\lambda_{i}\right)}}\right|$$

for identical acquisition parameters for Raman and SERDS $t_{SERDS}=2\cdot t_{RAMAN}$

(7) $$SNR_{RAMAN}\left(\lambda_{i}\right)=\frac{2\cdot S_{1}\left(\lambda_{i}\right)}{\sqrt{2}\cdot \sqrt{S_{1}\left(\lambda_{i}\right)+F_{1}\left(\lambda_{i}\right)}}$$

(8) $$\frac{SNR_{SERDS}\left(\lambda_{i}\right)}{SNR_{RAMAN}\left(\lambda_{i}\right)}=\frac{1}{2}\cdot \left|\frac{S_{1}\left(\lambda_{i}\right)-S_{2}\left(\lambda_{i}\right)}{S_{1}\left(\lambda_{i}\right)}\right|$$

(9) $$SNR_{SERDS}\left(\lambda_{i}\right)=\frac{SNR_{RAMAN}\left(\lambda_{i}\right)}{2}\cdot \left(\left|1-\frac{S_{2}\left(\lambda_{i}\right)}{S_{1}\left(\lambda_{i}\right)}\right|\right)$$

(10) $$SNR_{SERDS}=\frac{1}{2}\cdot \overset{n}{\underset{i=1}{\sum }}SNR_{RAMAN}\left(\lambda_{i}\right)\cdot \left(\left|1-\frac{S_{2}\left(\lambda_{i}\right)}{S_{1}\left(\lambda_{i}\right)}\right|\right)\;for\;S_{1}\geq 1$$

with *S*—Raman signal; *F*—fluorescence signal; $\lambda_{i}$—wavelength; *t*—acquisition time.

### 3.2. Experimental Approach

**Figure 4:**
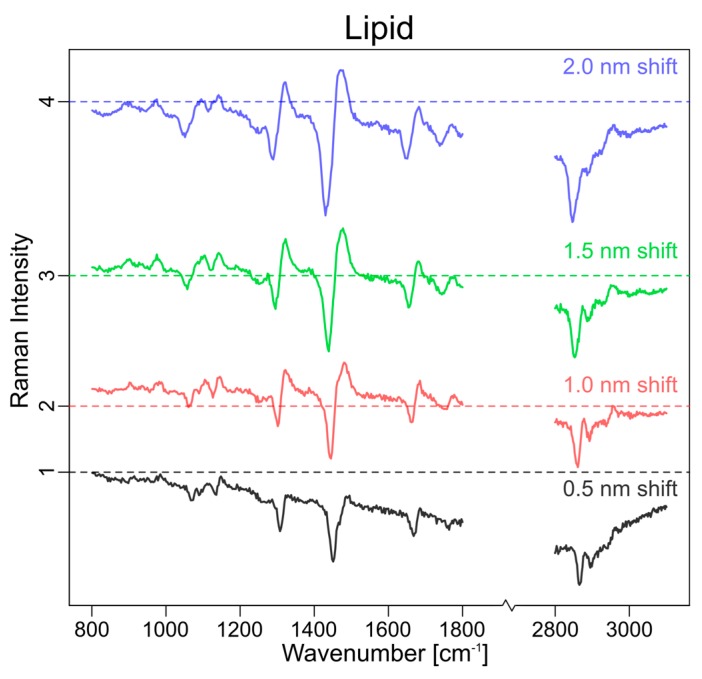
Measured difference mean raw spectra of a lipid cluster at different excitation wavelength shifts (shift: 0.5 nm (black), 1.0 nm (red), 1.5 nm (green), 2.0 nm (blue)).

Since the simulations showed the influence of the shift in excitation wavelength on the SERDS spectra, SERDS measurements of tissue samples were performed with different wavelength shifts. Because the largest shift that could be realized in our experimental setup was 2 nm (ca. 32 cm^−1^), four different shifts in excitation wavelength were measured in a 0.5 nm interval. In Figure 4 these different mean SERDS spectra of raw data for the lipid cluster of a tissue sample are shown. They show the same tendencies that could be seen in the simulations: the largest shift (blue) has the highest intensity, while the lowest can be observed for the smallest shift (black). This 0.5 nm shift is so small that the retained background is significant to the retained signal and cannot correct the spectra efficiently. It is also evident that the subtraction of raw spectra does not remove the background completely.

For the comparison of SERDS and EMSC, the largest possible shift was applied for the experimental measurements. Raw spectra (*λ*_1_ = 786 nm, *λ*_2_ = 784 nm, *λ_SERDS_* = *λ*_1_ − *λ*_2_) were used to generate the SERDS spectra. The EMSC correction was performed on the spectra measured at 785.0 nm.

**Figure 5:**
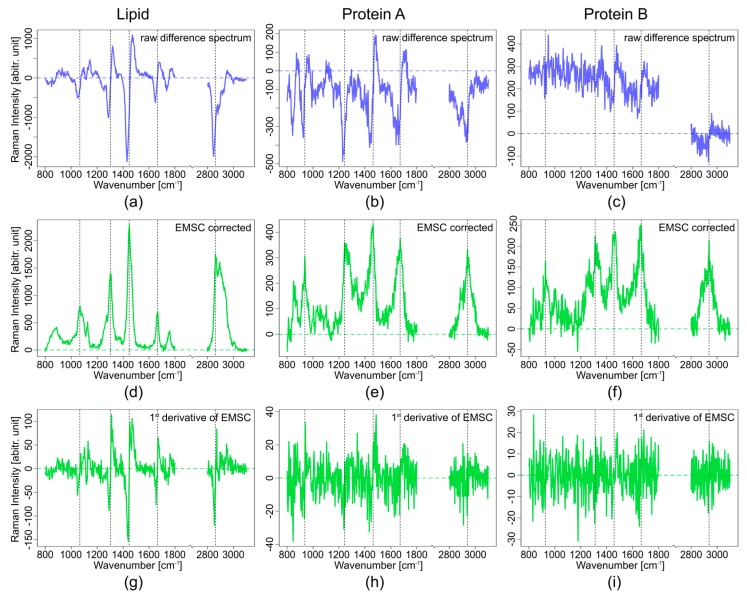
Comparison of measured lipid spectrum (**a**,**d**,**g**) and two protein spectra (**b**,**e**,**h**) and (**c**,**f**,**i**). Raw SERDS difference spectra (2 nm shift, (**a**–**c**), blue); EMSC corrected spectra at 785 nm ((**d**–**f**), green); 1st derivative of the EMSC corrected spectra at 785 nm ((**g**–**i**), green); the vertical black lines mark band positions.

The correction of real data with the computational background correction method EMSC can be demanding, if there are no pure component spectra available for the measuring system. For a system where all contributing spectral components are known this method works very well even for spectra with a high fluorescence background. The fluorescence background can be approximated by a higher polynomial and used as a background component. For a Raman image of a complex tissue with multiple spectra of different components, background estimation for fluorescence can be challenging since the contribution might vary locally. Even if multiple fluorescence background estimates are used for the EMSC algorithm, unknown varying backgrounds can cause performance problems for the EMSC background correction. On the other hand, SERDS difference spectra are hard to interpret since the band position cannot be reconstructed easily. Since difference spectra can be compared to the 1st derivative of a Raman spectrum, the 1st derivative of the EMSC corrected Raman spectra were calculated (Figure 5g–i). The black vertical lines show the band position in the EMSC corrected spectra (Figure 5d–f). For the lipid spectrum the band positions of the EMSC corrected spectrum overlaps quite nicely with the inflection points of the 1st derivative and the SERDS spectrum as seen in Figure 5a,d,g. For protein spectra, the band position of the EMSC corrected spectra also overlap with the inflection points of the 1st derivative of the EMSC and the SERDS spectra (Figure 5b,e,h and Figure 5c,f,i). However, the difference spectra are noisy and the bands are not easily located. It is even worse for the 1st derivative EMSC corrected spectra of proteins, which has a very high noise level. In the weaker protein spectrum in Figure 5c,f,i, bands are very hard to discern because of the high noise in the 1st derivative spectrum.

The intensity loss due to the subtraction of two raw spectra to generate the SERDS spectrum is clearly evident. The SERDS intensity is approximately half of the intensity of the EMSC spectra as seen in Figure 5. The SNR for the SERDS spectra is also lower than for the EMSC corrected spectra.

**Figure 6:**
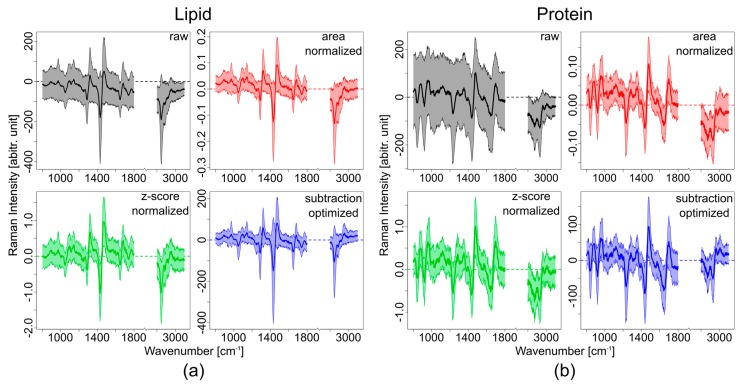
Comparison of different SERDS data processing methods with a 2 nm shift for (**a**) measured lipid spectra and (**b**) measured protein spectra. For every method the mean spectrum (darker shade) along with its standard deviation is shown. Black: difference spectra obtained by subtraction of raw data; red: difference spectra after area normalization; green: difference spectra after z-score normalization; blue: difference spectra after subtraction optimization.

Unlike in the simulated data the measured Raman spectra cannot be corrected by subtraction of one Raman spectrum from another. As shown in Figure 5 (blue spectra) and Figure 6a,b (black spectra), a simple subtraction of the raw spectra does not result in background-free difference spectra. For example, the mean spectrum of lipids is below zero intensity and has a bent in the spectral profile (Figure 6, black spectra). Photobleaching and variation in the laser power at the different excitation wavelengths cause changes in the background and total intensity, making the simple correction approach not suitable for most biological samples. A better overlap of the SERDS pair has to be accomplished, which requires further data processing before calculating the difference spectra.

Spectral normalization is one possible approach to obtain same relative intensities for all excitation wavelengths. Two different normalization procedures, as well as a subtraction optimization, were applied to the raw spectra as explained in the methods section. In Figure 4 the effect of the different spectral processing methods on lipid and protein spectra with fluorescence contributions are shown.

Lipids have a high Raman scattering cross-section and frequently occur at high local concentrations and therefore have a high Raman intensity as in comparison to protein for identical acquisition conditions, leading to strong difference bands. Due to the high intensity in comparison to the noise contribution, the noise-related standard deviation of the lipid difference spectra is low in comparison to protein spectra. Proteins have a smaller Raman scattering cross-section in comparison to lipids and have a lower local concentration, and a higher noise-related standard deviation in the protein difference spectra.

By applying area normalization to the SERDS spectra before subtraction, an improvement of the background correction can be observed. This improvement is a result of the better overlap between the spectra (Figure 6a,b, red spectra). There are still background contributions left in the difference spectra, which are clearly visible in the bending and the offset of the protein spectra (Figure 6b, red spectrum).

The z-score normalization results in an even better overlap between the spectra (Figure 6a,b, green spectra). The background in the difference spectra is minimized and the mean spectra are close to zero intensity for areas with no signal. Especially for the lipid spectra the z-score normalized difference spectrum has almost no background and no bending. For the high wavenumber region, however, this normalization also does not work well.

A further approach, which was tested, is based on subtraction optimization. Due to minimization of the area under the difference spectra, artifacts based on different laser intensities at the different excitation wavelength, and filter throughput of the different wavelength can be corrected. Still, after the subtraction optimization small background contributions are left (Figure 6a,b, blue spectra). Although the difference spectra are closer to zero where there is no signal, there is still a deviation visible especially in the protein difference spectrum. The intensity differences of the Raman bands in the difference spectrum are more pronounced than in the other methods and the standard deviation is minimized. The high wavenumber region is better corrected than for the other methods.

The results show that a simple subtraction of the raw spectra is not suitable to remove background contributions in a Raman spectrum with high auto-fluorescence contributions. With preprocessing of the spectra based on normalization or subtraction optimization a better overlap of the SERDS pair can be achieved. Nevertheless, some background artifacts still remain. Especially the difference spectra of the broad envelope of CH_n_ bands between 2800 and 3100 cm^−1^ result in not very pronounced difference bands. This shows that the small wavelength shift of 2 nm is not sufficient to have a clear separation of the broad CH_n_ bands. Hence, the CH_n_ bands are canceled out by subtracting the two spectra**.** Z-score normalization and the subtraction optimization both result in a better spectral overlap of the background and provide a better correction than the area normalization. The remaining background of the difference spectra can be corrected, using a polynomial fitting approach.

## 4. Discussion

The goal of this research was to compare an instrumental with a computational background correction method for Raman spectroscopy of biological samples. One of the most common instrumentation-based methods for background correction of Raman spectra is SERDS, which was compared to the computational method EMSC. The evaluation between the methods was first performed on simulated fluorescence and noise levels, and then on measured spectra.

The simulation of the effect of fluorescence and shot noise showed that Raman bands can be barely distinguished even if the fluorescence intensity is just five times higher than the maximal Raman band of the lipid signal, because of the shot noise resulting from fluorescence background. The SNR at each wavenumber of the simulated spectra was calculated and an estimated value was used to evaluate the overall SNR for different fluorescence signal intensities and difference spectra. This demonstrated that the SNR of the difference spectrum is lower than for a normal Raman spectrum, because the signal is subtractive while the noise is additive. It was also shown that the SNR is reduced inversely proportional to the square root of the fluorescence background for a Raman spectrum with fixed signal intensity. In contrary to previous suggestions to use very small shifts, which correspond to the full width at half maximum of a Raman band, a new criterion was proposed: i.e., the optimal shift is defined as the wavelength shift that retains the most information in the spectrum. By using the autocorrelation function, an optimal shift for lipids and proteins, using the proposed criterion was estimated. It was found that there is a region between 5 and 7.5 nm (for lipid) that retains the highest information and results in the highest SNR. This corresponds to a wavenumber shift of 80 to 120 cm^−1^. If the spectra are shifted by more than 7.5 nm, the correlation between the spectra will result in reduced signal after the subtraction. The reduction is due to an increase in an overlap between bands, which do not correspond to the same vibrations, being shifted into each other, resulting in misleading Raman difference information.

For EMSC it was shown that broad polynomial backgrounds could be removed without distorting the Raman bands and compromising the signal intensity nor adding additional noise to the spectra. Furthermore, EMSC can be superior to SERDS, since the spectral acquisition of SERDS requires the acquisition of two spectra, and hence, twice the acquisition time.

For the experimental implementation of SERDS a shift of 5 to 7.5 nm, as required for lipid spectra, can currently not be implemented in our setup, due to limits imposed by the used filters and the intensity stability of the excitation source. A wavelength shift of 2.0 nm was the largest implementable shift without a loss in excitation power. Still, the experimental data supported the simulated results, i.e., the larger the shift, the higher the retained intensities in SERDS spectra, which was shown experimentally for shifts of 0.5 nm, 1.0 nm, 1.5 nm and 2.0 nm for lipids, and the better the SNR. The experiments have shown that for protein and lipid spectra a simple subtraction of the shifted raw spectra, as is usually done for SERDS, does not suffice to obtain background-free SERDS spectra, and more complex computational methods, such as normalization or optimization, have to be used. From the three tested methods, z-score normalization and the subtraction optimization gave the best results. After performing EMSC on single Raman spectra, it was shown theoretically and experimentally that the EMSC corrected spectra can recover the main Raman bands for lipids and proteins. The simulation of the EMSC corrected spectra provided the same information as the original spectra, even when shot noise was added. In contrast to the SERDS difference spectra, the spectral interpretation of the Raman bands is not affected. To have a better comparison between difference spectra and EMSC corrected spectra, the 1st derivatives of the EMSC corrected spectra were calculated based on the experimental data set. It is clearly visible that SERDS experiences a high loss of intensity information due to the subtraction. Since for SERDS two Raman spectra have to be acquired, the measurement time is doubled compared to conventional Raman spectroscopy, leading to long acquisition times for Raman imaging of tissue samples. However, a drawback of the EMSC method is the estimation of very complex unknown backgrounds or multiple different fluorescence backgrounds, which can occur in a single Raman images, due to photobleaching or presence of different fluorophores in the sample.

## 5. Conclusions

For SERDS a reasonably good SNR is necessary to achieve a good background correction. The spectra have to be normalized or optimized before calculation of the SERDS spectra to compensate for fluctuations in signal intensity of the fluorescence background. Even then a further background correction is needed to obtain background-free difference spectra. The larger the shift of the excitation wavelength, the more signal intensity in a spectrum can be retained after the subtraction. For lipids the optimal shift is at 110 cm^−1^ (7 nm for 785 nm excitation) and for protein at 160 cm^−1^ (10 nm for 785 nm excitation). The proposed wavelength shifts are rather large, and can lead to changes in fluorescence intensity or, in the worst case, can excite different fluorophores and result in completely different fluorescence profiles. The interpretation of the difference spectra can be challenging, since it is hard to determine the exact band positions. On the other hand, SERDS is advantageous because no previous knowledge of the background is necessary for a correction.

Background correction with EMSC gives promising results for the simulated and the experimental data. It can, however, be challenging when dealing with complex samples and complex backgrounds. Pure spectra have to be generated and fluorescence background components have to be approximated. For Raman images with different fluorescence backgrounds, this can be tedious since more than one fluorescence component will be necessary.

In summary, SERDS and EMSC are both powerful tools for fluorescence background correction, with distinct advantages and disadvantages. When background spectra can be estimated, EMSC outperforms SERDS, because it keeps the fidelity of the Raman spectrum, does not require additional equipment, and guaranties a higher SNR. When, however, backgrounds are too complex to be estimated, SERDS could be a good choice as a background correction method.

## References

[1] Krafft C., Schie I., Meyer T., Schmitt M., Popp J. (2016). Development in spontaneous and coherent Raman scattering microscopic imaging for biomedical applications. Chem. Soc. Rev..

[2] Krafft C., Schmitt M., Schie I., Cialla-May D., Matthäus C., Bocklitz T., Popp J. (2017). Label-free molecular imaging of biological cells and tissues by linear and non-linear Raman spectroscopic approaches. Angew. Chem..

[3] Stone N. (2007). The use of Raman spectroscopy to provide an estimation of the gross biochemistry asociated with urological pathologies. Anal. Bioanal. Chem..

[4] Crow P., Uff J.A., Farmer J.A., Wright M.P., Stone N. (2004). The use of Raman spectroscopy to identify and characterize transitional cell carcicoma in vitro. BJU Int..

[5] Kerr L.T., Domikan K., Cullen I., Hennelly B.M. (2014). Applications of Raman spectroscopy to the urinary bladder for cancer diagnostics. Photonics Lasers Med..

[6] Crow P., Molckovsky A., Stone N., Uff J., Wilson B., Wongkeesong L.-M. (2005). Assesment of fiberoptic Near-infrared Raman spectroscopy for diagnostics of bladder and prostate cancer. J. Urol..

[7] Bergholt M.S., Zheng W., Ho K.Y., Teh M., Yeoh K.G., So J.B.Y., Shabbir A., Huang Z. (2014). Fiberoptic confocal Raman spectroscopy for real-time in vivo diagnosis of dysplasia in Barrett’s esophagus. Gastroenterology.

[8] Eberhardt K., Stiebing C., Matthäus C., Schmitt M., Popp J. (2015). Advantages and limitations of Raman spectroscopy for molecular diagnostics: An update. Expert Rev. Mol. Diagn..

[9] Motz J.T., Gandhi S.J., Scepanovic O.R., Feld M.S. (2005). Real-time Raman system for in vivo disease diagnosis. J. Biomed. Opt..

[10] Draga R.O.P., Grimbergen M.C.M., Vijverberg P.L.M., Bosch J.L.H.R. (2010). In Vivo Bladder Cancer Daignosis by High-Volume Raman Spectroscopy. Anal. Chem..

[11] De Jong B.W.D., Schut T.C.B., Wolffenbuttel K.P., Nijman J.M., Kok D.J., Puppels G.J. (2002). Identification of bladder wall layers by Raman spectroscopy. J. Urol..

[12] Monici M. (2005). Cell and tissue autofluorescence research and diagnostic applications. Biotechnol. Annu. Rev..

[13] McCain S.T., Willett R.M., Brady D.J. (2008). Multi-excitation Raman Spectroscopy Technique for Fluorescence Rejection. Opt. Express.

[14] Macdonald A.M., Wyeth P. (2006). On the use of photobleaching to reduce fluorescence background in Raman spectroscopy to improve the reliability of pigment identification on painted textiles. J. Raman Spectrosc..

[15] Zięba-Palus J., Michalska A. (2014). Photobleaching as a useful technique in reducing of fluorescence in Raman spectra of blue automobile paint samples. Vib. Spectrosc..

[16] Da Silva M.A., Riveiro D.G., Pereira E.A., Martin A.A., Fontes A., da Silva Martinho H. (2017). Shifted-excitation difference spectroscopy for in vitro and in vivo biological samples analysis. Biomed. Opt. Express.

[17] Zhang Z.-M., Chen S., Liang Y.-Z., Liu Z.-X., Zhang Q.-M., Ding L.-X., Yec F., Zhou H. (2009). An intelligent background-correction algorithm for highly fluorescent samples in Raman spectroscopy. J. Raman Spectrosc..

[18] Venables B., Hornik K., Maechler M. (2014). Polynom: A Collection of Functions to Implement a Class for Univariate Polynomial Manipulations. https://rdrr.io/github/eestileib/ComplexPoly/.

[19] Kourkoumelis N., Polymeros A., Tzaphlidou M. (2012). Background estimation of biomedical Raman spectra using a geometric approach. Spectrosc. Int. J..

[20] Gautam R., Vanga S., Ariese F., Umapathy S. (2015). Review of multidimensional data processing approaches for Raman and infrared spectroscopy. EPJ Tech. Instrum..

[21] Lieber C.A., Mahadevan-Jansen A. (2003). Automated Method for Subtraction of Fluorescence from Biological Raman Spectra. Appl. Spectrosc..

[22] Cadusch P.J., Hlaing M.M., Wade S.A., MxArthur S.L., Stoddart P.R. (2013). Improved methods for fluorescence background substraction from Raman spectra. Mater. Sci..

[23] Martens H., Nielsen J.P., Engelsen S.B. (2003). Light scattering and ligth absorbance separated by extended multiplicative signal correction. Application to near-infrared transmission analysis of powder mixtures. Anal. Chem..

[24] Mecozzi M. (2014). A Polynomial Curve Fitting Method for Baseline Drift Correction in the Chromatographic Analysis of Hydrocarbons in Environmental Samples. APCBEE Procedia.

[25] Massart D.L., Vandeginste B.G.M., Buydens L.C.M., Jong S.D., Lewi P.J., Smeyers-Verbeke J. (1997). Data Handling in Science and Technology, Handbook of Chemometrics and Qualimetrics, Vols. 20A and 20B.

[26] Vinzi V.E., Chin W.W., Henseler J., Wang H. (2010). Handbook of Partial Least Squares: Concepts, Methods and Applications.

[27] Baek S.J., Park A., Ahn Y.J., Choo J. (2014). Baseline correction using asymmetrically reweighted penalized least squares smoothing. Analyst.

[28] Liland K.H., Kohler A., Afseth N.K. (2015). Model-based pre-processing in Raman spectroscopy of biological samples. J. Raman Spectrosc..

[29] Hasegawaa T., Nishijoa J., Umemurab J. (2000). Separation of Raman spectra from fluorescence emission background by principal component analysis. Chem. Phys. Lett..

[30] Asfour H., Swift L.M., Sarvazyan N., Doroslovacki M., Kay M.W. (2011). Signal Decomposition of Transmembrane Voltage-Sensitive Dye Fluorescence Using a Multiresolution Wavelet Analysis. IEEE Trans. Biomed. Eng..

[31] Qu H.-B., Ou D.-L., Cheng Y.-Y. (2015). Background correction in near-infrared spectra of plant extracts by orthogonal signal correction. J. Zhejiang Univ. Sci. B.

[32] Dennis A. (2007). Photo-Bleaching and Automatic Baseline Correction for Raman Spectroscopy.

[33] Knorr F., Smith Z.J., Wachsmann-Hogiu S. (2010). Development of a time-gated system for Raman spectroscopy of biological samples. J. Opt. Soc. Am..

[34] Shreve A.P., Cherepy N.J., Mathies R.A. (1992). Effective Rejection of Fluorescence Interference in Raman Spectroscopy Using a Shifted Excitation Difference Technique. Spectrosc. Tech..

[35] De Luca A.C., Mazilu M., Riches A., Herrington C.S., Dholakia K. (2010). Online Fluorescence Suppression in Modulated Raman Spectroscopy. Anal. Chem..

[36] Dongy S., Melnik E.D., Ban V.S., Volodin B.L. (2012). A novel Method for practical implementation of shifted-excitation Raman difference spectroscopy (SERDS). Spectroscopy.

[37] Gebrekidan M.T., Knipfer C., Stelzle F., Popp J., Will S., Braeuer A. (2015). A shifted-excitation Raman difference spectroscopy (SERDS) evaluation strategy for the efficient isolation of Raman spectra from extreme fluorescence interference. J. Raman Spectrosc..

[38] Osticioli I., Zoppi A., Castellucci E.M. (2007). Shift-Excitation Raman difference spectroscopy-difference deconvolution methods for the luminescence background rejection from Raman spectra of solid samples. Appl. Spectrosc..

[39] Oshima Y., Komachi Y., Furihata C., Tashiro H., Sato H. (2006). Fluorescence-suppressed Raman technique for quantitative analysis of protein solution using a micro-Raman probe, the shifted excitation method, and partial least squares regression analysis. Appl Spectrosc..

[40] Dochow S., Ma D., Latka I., Bocklitz T., Hartl B., Bec J., Fatakdawala H., Marple E., Urmey K., Wachsmann-Hogiu S. (2015). Combined fiber probe for fluorescence lifetime and Raman spectroscopy. Anal. Bioanal. Chem..

[41] Zhao J., Short M., Braun T., Lui H., McLean D., Zeng H. (2014). Clinical Raman measurements under special ambient lighting illumination. J. Biomed. Opt..

[42] Maiwald M., Müller A., Sumpf B., Erbert G., Tränkle G. (2015). Capability of shifted excitation Raman difference spectroscopy under ambient daylight. Appl. Opt..

[43] Zhao J., Carrabba M.M., Allen F.S. (2002). Automated Fluorescence Rejection Using Shifted Excitation Raman Difference Spectroscopy. Appl. Spectrosc..

[44] Dochow S., Bergner N., Krafft C., Clement J., Mazilu M., Praveen B.B., Ashok P.C., Marchington R., Dholakia K., Popp J. (2013). Classification of Raman spectra of single cells with autofluorescence suppression by wavelength modulated excitation. Anal. Methods.

[45] Mazilu M., Luca A.C.D., Riches A., Herrington C.S., Dholakia K. (2010). Optimal algorithm for fluorescence suppression of modulated Raman spectroscopy. Opt. Express.

[46] Krafft C., Dochow S., Bergner N., Clement J.H., Praveen B.B., Mazilu M., Marchington R., Dholakia K., Popp J. Raman spectra of single cells with autofluorescence suppression by modulated wavelength excitation. Proceedings of the Biomedical Vibrational Spectroscopy V: Advances in Research and Industry.

[47] Schmidt H., Sowoidnich K., Kronfeldt H.-D. (2010). A Prototype Hand-Held Raman Sensor for the in situ Characterization of Meat Quality. Appl. Spectrosc..

[48] Bell S.E.J., Bourguignon E.S.O., Dennis A. (1998). Analysis of luminescent samples using subtracted shifted Raman spectroscopy. Analyst.

[49] Matousek P., Towrie M., Parker A.W. (2005). Simple reconstruction algorithm for shifted excitation Raman difference spectroscopy. Appl. Spectrosc..

[50] Willett R. Multiscale reconstruction for photon-limited shifted excitation Raman spectroscopy. Proceedings of the IEEE International Conference on Acoustics, Speech and Signal Processing—ICASSP.

[51] Schmidt H., Kaiser D.P., Maiwald M. (2012). Method for Generating and for Detecting a Raman Spectrum. U.S. Patent.

[52] Team R.C. (2016). R: A Language and Environment for Statistical Computing.

[53] Beleites C., Sergo V. (2015). HyperSpec: A Package to Handle Hyperspectral Data Sets in R.

[54] Liland K.H., Mevik B.-H. (2015). Baseline:Baseline Correction of Spectra. https://cran.r-project.org/web/packages/baseline/index.html.

[55] Borchers H.W. (2017). Pracma: Practical Numerical Math Functions. https://www.rdocumentation.org/packages/pracma/versions/1.9.9.

[56] Beleites C. (2013). Ramancal: Calibration Routines for Raman Spectrometers.

[57] Hyndman R. (2016). (Partial) Autocorrelation and Cross Correlation Function Estimation. https://www.rdocumentation.org/packages/forecast/versions/7.3.

[58] Hyndman R.J. (2017). Forecast: Forecasting Functions for Time Series and Linear Models. https://rdrr.io/cran/forecast/.

[59] Beleites C. (2016). Cbmodels: Collection of “Combined” Models: PCA-LDA, PLS-LDA, PLS-LR as Well as EMSC.

[60] McCreery R.L. (2000). Raman Spectroscopy for Chemical Analysis.

[61] Kiselev R., Schie I.W., Askrabic S., Krafft C., Popp J. (2016). Design and first applications of a flexible Raman micro-spectroscopic system for biological imaging. Biomed. Spectrosc. Imaging.

[62] Ryabchykov O. (2016). Spikes: Spike Correction of Raman Spectral Data.

[63] Ryabchykov O., Bocklitz T., Ramoji A., Neugebauer U., Foerster M., Kroegel C., Bauer M., Kiehntopf M., Popp J. (2016). Automatization of spike correction in Raman spectra of biological samples. Chemom. Intell. Lab. Syst..

[64] Dhanoa M.S., Barnes R.J., Lister S.J. (1989). Standard normal variate transformation and de-trending of near-infrared diffuse reflectance spectra. Appl. Spectrosc..

[65] Slobodan S. (2008). Pharmaceutical Applications of Raman Spectroscopy.

[66] Long D.A. (2002). The Raman Effect: A Unified Treatment of the Theory of Raman Scattering by Molecules.

[67] Maiwald M., Müller A., Sumpf B., Tränkle G. (2016). A portable shifted excitation Raman difference spectroscopy system: Device and field demonstration. J. Raman Spectrosc..

[68] De Luca A.C., Dholakia K., Mazilu M. (2015). Modulated Raman Spectroscopy for Enhanced Cancer Diagnosis at the Cellular Level. Sensors.

